# Late Local Recurrence of Bone Giant Cell Tumors Associated with an Increased Risk for Malignant Transformation

**DOI:** 10.3390/cancers13143644

**Published:** 2021-07-20

**Authors:** Shinji Tsukamoto, Alberto Righi, Andreas F. Mavrogenis, Manabu Akahane, Kanya Honoki, Yasuhito Tanaka, Davide Maria Donati, Costantino Errani

**Affiliations:** 1 Department of Orthopaedic Surgery, Nara Medical University, Kashihara-City 634-8521, Japan; kahonoki@naramed-u.ac.jp (K.H.); yatanaka@naramed-u.ac.jp (Y.T.); 2 Department of Pathology, IRCCS Istituto Ortopedico Rizzoli, 40136 Bologna, Italy; alberto.righi@ior.it; 3 First Department of Orthopaedics, School of Medicine, National and Kapodistrian University of Athens, 15562 Athens, Greece; afm@otenet.gr; 4 Department of Health and Welfare Services, National Institute of Public Health, Saitama 351-0197, Japan; akahane.m.aa@niph.go.jp; 5 Department of Orthopaedic Oncology, IRCCS Istituto Ortopedico Rizzoli, 40136 Bologna, Italy; davide.donati@ior.it (D.M.D.); costantino.errani@ior.it (C.E.)

**Keywords:** giant cell tumor of bone, denosumab, surgery, metastasis, malignant transformation, recurrence, chemotherapy

## Abstract

**Simple Summary:**

In giant cell tumor of bone (GCTB), an intermediate malignant bone tumor, approximately 4% of cases can undergo malignant transformation. We analyzed risk factors for malignant transformation of GCTB treated without radiotherapy and retrospectively reviewed medical files of 461 patients with GCTB of the extremities who had undergone surgery alone, with no radiotherapy or denosumab therapy. Malignant transformation occurred in 15 of 461 patients (3.3%) at a median follow-up period of 192 months. The median follow-up duration was 89.4 months. Multivariate analysis revealed that local recurrence was an independent prognostic factor for unfavorable malignant transformation. The interval between the last surgery to local recurrence and malignant transformation was longer than that to local recurrence of benign GCTB, with a median of 15.2 (IQR, 5.2–25.4) years versus 1.3 (IQR, 0.8–2.6) months, respectively (*p* < 0.001). Late local recurrence of GCTB is associated with a higher risk of malignant transformation.

**Abstract:**

In giant cell tumor of bone (GCTB), an intermediate malignant bone tumor, approximately 4% of all cases undergo malignant transformation. Accordingly, we analyzed risk factors for malignant transformation of GCTB treated without radiotherapy. We retrospectively reviewed medical records of 530 patients with GCTB of the extremities, admitted and treated at two institutions between January 1980 and December 2019. Overall, 4 patients with primary malignant GCTB, 4 patients with missing data, 3 patients with a history of radiotherapy, 22 patients with a follow-up of less than 6 months, and 36 patients who received denosumab were excluded. Accordingly, 461 patients were included for further analysis. Malignant transformation was observed in 15 of 461 patients (3.3%) at a median follow-up period of 192 months. The median follow-up duration was 89.4 months. Multivariate analysis revealed that local recurrence was an independent prognostic factor for unfavorable malignant transformation (Hazard ratio [HR], 11.33; 95% confidence interval [CI] 2.33–55.13; *p* = 0.003 for once versus none and HR, 11.24; 95% CI, 1.76–71.96; and *p* = 0.011 for twice or more versus none). The interval between the last surgery to local recurrence and malignant transformation was longer than that to local recurrence of benign GCTB, with a median of 15.2 years (interquartile range [IQR], 5.2–25.4) versus 1.3 months (IQR, 0.8–2.6), respectively (*p* < 0.001). Late local recurrence of GCTB is associated with a higher risk of malignant transformation.

## 1. Introduction

Giant cell tumor of bone (GCTB) is an intermediate malignant osteoclastogenic stromal tumor with a broad biological spectrum [1]. Genetically, GCTB is characterized by specific mutations in the H3F3A gene, which encodes histone H3.3 [2]. Typically, this tumor involves the metaphyseal-epiphyseal region of long bones [3]. Furthermore, no gender-based predilection has been noted, and the peak incidence is between 20 and 45 years of age [4,5]. Curettage is followed by minimal disability but may be associated with a relatively high local recurrence rate [4,5,6]. Resection has been associated with a lower risk of local recurrence but can lead to relatively severe functional impairment [5].

Primary and secondary malignant GCTBs account for approximately 4% of all GCTB cases [7,8]. Primary malignant GCTB is simultaneously diagnosed with sarcoma during the initial GCTB diagnosis. Secondary malignant GCTB occurs when the malignancy is diagnosed at the site of a GCTB that is previously treated with surgery or radiotherapy [7]. Radiotherapy reportedly induces late malignant transformation of GCTB [9,10,11,12,13] and is usually not recommended for treating GCTB [8]. Typically, patients with GCTB are relatively young. Misdiagnosis of malignant GCTB significantly worsens patient prognosis, as wide resection, with or without chemotherapy, is required to treat secondary malignant GCTB [7]. Therefore, it is crucial to consider risk factors for malignant transformation during the follow-up of patients with GCTB. However, there are no available reports on the risk factors associated with the malignant transformation of GCTB. Accordingly, we performed a retrospective assessment to analyze risk factors for malignant transformation of GCTB treated without radiotherapy.

## 2. Patients and Methods

### 2.1. Patient Selection

We retrospectively reviewed the medical records of 530 patients with GCTB of the extremities who had been admitted and treated at two institutions (IRCCS Istituto Ortopedico Rizzoli and Nara Medical University) between January 1980 and December 2019. IRCCS Istituto Ortopedico Rizzoli is a high-volume center specializing in bone and soft tissue tumors that is referred from all over Italy. Nara Medical University is a certified institute specializing in the treatment of sarcoma of the extremities, which is a tertiary hospital in Nara prefecture, Japan. Inclusion criteria included patients with GCTB of the extremities who had a postoperative follow-up period of 6 months or longer. Exclusion criteria included patients with primary malignant GCTB and missing data, patients with a history of radiotherapy owing to the possible relationship between radiotherapy and malignant transformation [8], and patients who received denosumab for GCTBs, owing to the possible association between denosumab administration and malignant transformation [14,15,16,17,18,19,20,21,22,23,24,25]. We retrieved the following data from the patients’ medical records: age, sex, site, Campanacci stage of GCTB [4], previous surgery (curettage or en-bloc resection in another hospital), surgery type, local recurrence, lung metastasis (synchronous or metachronous), malignant transformation, and follow-up period.

### 2.2. Statistical Analyses

Statistical differences between two independent samples were analyzed using the Mann–Whitney U test for nonparametric analyses. Malignant transformation was diagnosed when the malignant component of a GCTB was histologically observed following treatment of a benign GCTB [1]. Secondary malignant GCTBs are challenging to distinguish from primary sarcomas based on histology. Accordingly, clinical history and a pathological diagnosis of benign GCTB, as well as previous treatment, are crucial for diagnosis [7]. Malignant transformation-free survival was defined as the time from the date of initial surgical treatment of GCTB at our institute to the date of malignant transformation diagnosis or the last follow-up. The date of malignant transformation was defined as the date of pathological diagnosis of malignant transformation. Malignant transformation-free survival was evaluated using Kaplan–Meier survival analysis, and survival curves were compared using a log-rank test. Cox proportional-hazards regression analysis estimated the hazard ratios (HR) for malignant transformation risk factors. Statistical significance was set at *p* < 0.05. Analyses were performed using IBM SPSS (version 25.0; IBM Co., Armonk, NY, USA) and JMP 14 (SAS Institute Inc., Cary, NC, USA).

The independent ethics committee of each institution approved the study. Informed consent was obtained from all individual participants in IRCCS Istituto Ortopedico Rizzoli, and a waiver of informed consent from participants in Nara Medical University was provided.

## 3. Results

### 3.1. Patient Data and Treatment

**Figure 1:**
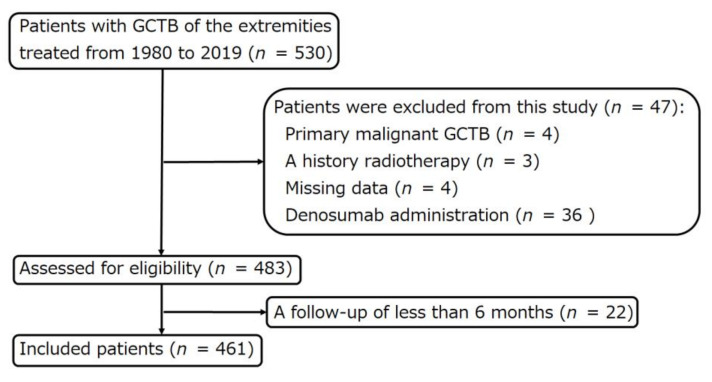
Flow diagram of patients with giant cell tumor of bone of the extremity treated at two institutions between 1980 and 2019.

Four patients with primary malignant GCTB and four patients with missing data were excluded from the analysis. Three patients with a history of radiotherapy were also excluded. In addition, 22 patients with a follow-up period of less than 6 months were excluded. Thirty-six patients who received denosumab preoperatively and postoperatively were excluded. The remaining 461 patients were included in this study for further analysis (Figure 1). No patient was recalled for the purpose of this study.

Curettage was indicated for patients with GCTB in Campanacci stages 1 and 2, with or without a pathological fracture [26,27]. Curettage was performed through a large cortical bone window using sharp curettes, enabling the removal of all visible tumor tissues [26,27]. The cavity was then curetted with a high-speed burr and washed with saline to remove all pathological tissues [26,27]. Subsequently, the tumor cavity was filled with bone allograft, polymethylmethacrylate bone cement, or hydroxyapatite [26,27]. En-bloc resection was indicated for patients with GCTB in Campanacci stage 3 or pathological fractures with joint invasion and GCTB in expendable bones [26]. Reconstruction after resection was performed using a modular prosthesis, massive bone allografts, allograft composite prostheses, or vascularized fibular autografts [26,27]. Amputation was indicated in four patients with an extensive tumor encasing the neurovascular bundle and precluding neurovascular bypass and limb-salvage surgery.

As shown in Table 1, the median age of patients was 30 years and the male–female ratio was 49% vs. 51%. Tumor sites were most common in the distal femur (33%), followed by the proximal tibia (26%), and the distal radius (12%). The Campanacci stage was 2% for stage 1, 58% for stage 2, and 40% for stage 3. The proportion of patients who had been treated at a previous hospital was 17%. Regarding surgical methods, curettage accounted for 59%, and resection and amputation accounted for 41%. Patients who had one local recurrence accounted for 14%, and those who had two or more local recurrences accounted for 4%. Patients with lung metastases at presentation accounted for 2%, and patients with lung metastases later accounted for 5%. Details of the 26 patients who experienced lung metastases without malignant transformation were shown in Supplementary File (Table S1).

### 3.2. Incidence of Malignant Transformation of GCTB

Malignant transformation occurred in 15 of 461 patients (3.3%) at a median of 192 months (IQR, 78–316) of follow-up (Table 2 and Table 3). Out of the 414 patients at IRCCS Istituto Ortopedico Rizzoli, 13 experienced malignant transformation. Out of the 47 patients at Nara Medical University, two experienced malignant transformation. The median follow-up was 89.4 months (IQR, 54.8–134.0) (Table 1). Local recurrence was detected in 83 of the 461 (18.0%) patients (Table 1).

### 3.3. Histology of Secondary Malignant GCTB

**Figure 2:**
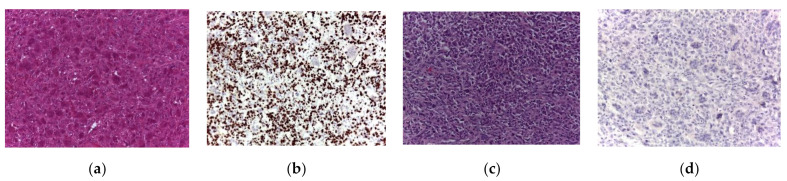
Histological specimens relative to secondary malignancy in giant cell tumor of bone. (**a**,**b**) Biopsy of the initial lesion, with the appearance of a giant cell tumor of bone (Hematoxylin and eosin [H&E], **a**, 200× magnification) with strong immunohistochemical expression of the H3F3A protein G34W variant in mononuclear cells (**b**, 200× magnification). (**c**,**d**) Biopsy of the recurrent lesion. Highly malignant cells with a giant cell component and focal osteoid production were present (H&E, **c**, 200× magnification). The spindled and pleomorphic neoplastic cells were negative for the H3F3A protein G34W variant (**d**, 200× magnification).

**Figure 3:**
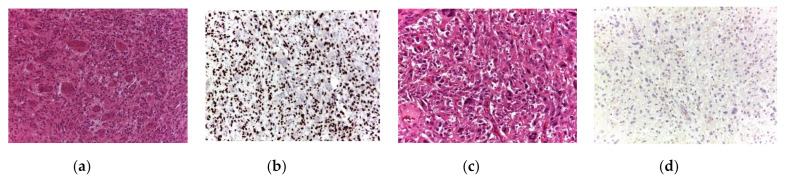
Histological specimens relative to secondary malignancy in giant cell tumor of bone. (**a**) Histology of the initial biopsy showing multinucleated giant cells embedded in oval, round mononuclear cells with hemosiderotic deposits; the lesion presents the appearance of a classic giant cell tumor (H&E, **a**, 200× magnification), confirmed by immunohistochemistry to express the H3F3A protein G34W variant in mononuclear cells (**b**, 200× magnification). (**c**) Biopsy of the recurrent lesion, where a highly malignant neoplasm constituting spindled and pleomorphic cells can be observed. Upon immunohistochemistry analysis, the H3F3A protein G34W variant was lost (**d**, 200× of magnification).

Based on histological analysis, secondary malignant GCTB was identified as osteosarcoma (10 patients; Figure 2a–d) and undifferentiated pleomorphic sarcoma (5 patients; Figure 3a–d). Of the 15 malignant GCTBs, 13 were negative for H3F3A mutation; however, two were positive.

### 3.4. Comparison of Time to Local Recurrence between Benign GCTB and Secondary Malignant GCTB

**Figure 4:**
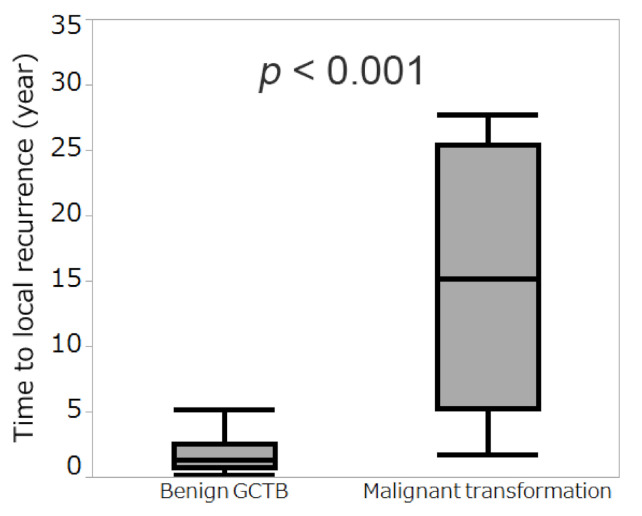
Mann-Whitney U test showing a significant difference between time to local recurrence of benign giant cell tumor of bone (GCTB) and time to local recurrence with malignant transformation (*p* < 0.001).

In 70 patients with local recurrence without malignant transformation, the median time to first local recurrence was 1.3 years (IQR, 0.8–2.6). In contrast, among the 12 patients diagnosed with local recurrence and malignant transformation, the median time from the last surgery to local recurrence with malignant transformation was 15.2 years (IQR, 5.2–25.4). Accordingly, a difference in the time to local recurrence was observed between groups (*p* < 0.001; Figure 4).

### 3.5. Outcomes in Patients Who Experienced Malignant Transformation

Four patients presented lung metastases at the time of malignant transformation diagnosis: two patients died due to the disease two and three months after diagnosis, and two patients survived at a nine-month follow-up after diagnosis. Eleven patients did not have distant metastases when malignant transformation was diagnosed, four patients underwent surgery only (one of these four patients died of their disease due to lung metastases), while seven patients underwent surgery and received adjuvant chemotherapy (none of these seven patients died of their disease). The median follow-up of patients with secondary malignant GCTB was 33 months (IQR, 9–97) (Table 2 and Table 3).

### 3.6. Risk Factors for Malignant Transformation from GCTB Treated without Radiotherapy

**Figure 5:**
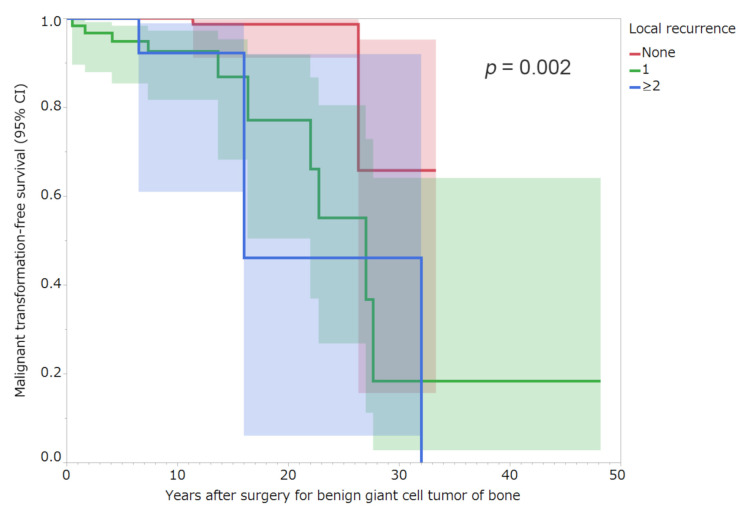
Malignant transformation-free survival rates of patients by the number of local recurrences. Shading around the curves represents the 95% confidence intervals (CI).

Upon univariate analysis, patients who had undergone curettage (10-year malignant transformation-free survival 97.8% [95% CI: 94.2–99.2]) presented a higher risk for malignant transformation when compared with patients who had undergone resection or amputation (99.2% [95% CI: 94.3–99.9]; *p* = 0.042; Table 4). Patients with local recurrence (10-year malignant transformation-free survival 92.4% [95% CI: 81.2–97.1] once, 92.3% [95% CI, 60.9–98.9] twice or more) had a higher risk of malignant transformation than those who did not present with local recurrence (100%; *p* = 0.002; Figure 5; Table 4). Univariate analysis revealed no association between the following variables and malignant transformation: age, sex, site, Campanacci stage, previous surgery, surgical method, and lung metastasis (Table 4). A stepwise multivariate analysis that included clinical variables related to unfavorable malignant transformation in the univariate analysis revealed that local recurrence was an independent risk factor for unfavorable malignant transformation (HR 11.33 [95% CI: 2.33–55.13]; *p* = 0.003 for once versus none, HR 11.24 [95% CI: 1.76–71.96]; *p* = 0.011 for twice or more versus none; Table 5).

### 3.7. Details of Excluded Patients Who Received Surgery and Denosumab

The details of the 36 patients receiving denosumab are presented in Supplementary File (Table S2). Denosumab was indicated for the downstaging of GCTBs located at the distal radius, as tumors at this location are considerably aggressive, and resection was associated with worse functional outcomes [26,28]. In addition, denosumab was prescribed for GCTBs where surgery could potentially result in severe morbidity [29]. In 36 patients, preoperative denosumab was administered subcutaneously at a dose of 120 mg once per week for 1 month, and then once per month for 2–30 months, based on the recommendation for discontinuation by treating physician, the occurrence of an adverse event, clinical benefit from treatment, surgical planning, or as per the clinical trial protocol. In 35 of the 36 patients, surgery was performed 1 month after the last preoperative denosumab administration, as follows: curettage in 30 patients and en-bloc resection in 5 patients. Only one patient did not undergo surgery and continued denosumab therapy for 29 months. The patient experienced local recurrence and underwent amputation three years after denosumab treatment was discontinued. In addition, the patient experienced lung metastasis and was diagnosed with malignant transformation 2 years and 10 months after amputation (Case 16; Table S3). Postoperative denosumab was administered at the same dose as that administered preoperatively in 25 of 30 patients who underwent curettage; in three of five patients who underwent en-bloc resection, denosumab was administered once per month for 1–6 months, depending on the recommendation for discontinuation by the treating physician [30]. In patients treated with denosumab, malignant transformation was detected in two of 36 patients (5.6%) at 48 and 100 months of follow-up. The median follow-up of 36 patients was 97.5 months (interquartile range [IQR], 84.8–109).

## 4. Discussion

Local recurrence of GCTB usually occurs within 2 years, therefore local recurrence after 2 years or more is considered to be “late” local recurrence [26]. In the present study, our findings revealed that “late” local recurrence is a risk factor for malignant transformation of GCTB of the extremities without a history of radiotherapy. Therefore, late local recurrence of GCTB should direct suspicion toward the risk of possible malignant transformation. Based on a case series of 20 patients with secondary malignant GCTB [7], core biopsies identified malignancy in 10 patients prior to definitive surgery; in the other 10 patients, malignancy was diagnosed following surgery to manage a local recurrence. It is difficult to differentiate secondary malignant GCTB from recurrent benign GCTB radiologically [7]. Herein, our findings revealed that the interval from the last surgery to local recurrence with malignant transformation was longer than that for local recurrence of benign GCTB. In addition, Liu et al. [7] have reported that the interval between local recurrence and malignant transformation was longer than that between local recurrence of benign GCTB (median 57 vs. 19 months). Moreover, the authors reported that an interval of 49.5 months between surgery and local recurrence was a critical threshold for distinguishing malignant transformation from recurrence of benign GCTB [7]. Our data were in line with these previous results, demonstrating that “late” local recurrence is associated with malignant transformation of GCTB. Thus, malignant transformation should be suspected, and biopsies for recurrent GCTB should be considered when the interval between the last surgery and local recurrence is more than two years (“late” local recurrence).

In the present study, two of four patients with secondary malignant GCTB who presented distant metastasis at the time of diagnosis died during the disease course. Our results revealed that patients with secondary malignant GCTB metastasis at presentation had a poor prognosis. A study using the Surveillance, Epidemiology, and End Results database found that older age, larger tumor size, regional or distant metastasis, and lack of radiotherapy were associated with poor overall survival in patients with both primary and secondary malignant GCTB [31]. Herein, among the 11 patients with localized secondary malignant GCTB, none died, owing to the disease course following surgery with adjuvant chemotherapy (seven patients), while one of four patients in the surgery alone group died due to the disease. Anract et al. [32] have reported improved 1-year survival in patients who underwent surgery with adjuvant chemotherapy when compared with those who received surgery alone; however, this benefit was not observed for 5-year survival. In addition, the authors reported that resection specimens from three of four patients with malignant GCTB, who had received neoadjuvant chemotherapy, showed a tumor response [32]. Liu et al. [7] have observed no benefit in overall survival in patients treated with adjuvant chemotherapy; however, adjuvant chemotherapy benefited lung metastasis-free survival. The 5-year survival rates in the chemotherapy and non-chemotherapy groups were 57.0% and 33.3%, respectively (*p* = 0.167) [7]. Median pulmonary metastasis-free survival in patients who received chemotherapy was significantly longer than in patients who underwent surgery alone (13 vs. 6 months) [7]. Our data did not support the efficacy of chemotherapy for malignant GCTB, but the small sample size could have induced a bias; hence, our results should be cautiously considered.

The efficacy and safety of denosumab for GCTB treatment have been reported, and the U.S. Food and Drug Administration approved the use of denosumab in 2013 [14]. However, 12 cases of malignant transformation of GCTB during and after denosumab treatment have been reported [14,15,16,17,18,19,20,21,22,23,24], suggesting that denosumab treatment is associated with malignant transformation [33]. According to recent systematic reviews, the cumulative incidence of secondary malignant GCTB without a history of radiotherapy or denosumab treatment was 0.6% [8]. Chawla et al. [33] have reported that malignant transformation occurred in four of 526 patients with GCTB (0.8%) after a median follow-up of 58 months post-denosumab treatment. In four patients, the time from GCTB diagnosis to malignant transformation ranged between 17 months and 11 years [34]. In addition, Chawla et al. [33] have reported that the frequency of confirmed malignant transformation in patients receiving denosumab treatment was similar to that observed in patients treated without denosumab. Agarwal et al. [16] have observed that malignant transformation occurred in 1 of 25 patients (4%) after a median follow-up of 27 months after denosumab was administered for 8 months postoperatively. Treffel et al. [23] have revealed that, among 35 patients who presented with GCTB and received denosumab treatment, malignant transformation occurred in one patient (2.9%), 18 months postoperatively. Recently, Perrin et al. [21] have reported that malignant transformation occurred in one of 25 patients (4%) with GCTB at a median follow-up of 57 months after denosumab administration, 55 months postoperatively. Accordingly, a longer follow-up duration is needed to confirm the safety of denosumab treatment for GCTB. Our data support these previous results by demonstrating that, after a median follow-up of 97.5 months after denosumab administration, malignant transformation occurred in two of 36 patients (5.6%) with GCTB, 48 and 100 months postoperatively.

Our study has several limitations. First, the median follow-up for all patients (89.4 months) was shorter than the median time to malignant transformation (192 months). Therefore, this study can only assess risk factors for relatively early malignant transformation. Second, stepwise multivariate analysis revealed that local recurrence was associated with malignant transformation. However, the number of patients with malignant transformation in this analysis was small. Multicenter collaborative studies, allowing increased data collection, will be crucial in the future. Third, of the entire set of 461 primary GCTB, 102 (22%) were tested for the presence of H3F3A mutation and all these 102 cases revealed a H3F3A mutation [35,36,37]. The remaining cases were not molecularly confirmed with the H3F3A mutation, as these patients were diagnosed before this assessment was introduced. However, these cases were diagnosed by experienced pathologists specializing in bone tumor pathology.

## 5. Conclusions

In conclusion, late local recurrence of GCTB is associated with a higher risk of malignant transformation. Therefore, special attention must be paid when a patient with GCTB presents with local recurrence after a long interval following primary tumor surgery.

## Figures and Tables

**Table 1:** Characteristics of patients included in this study.

Variable (*n* = 461)	Number of Patients
Age (Years)	
Median	30.0
Interquartile Range	23.3–41.6
Sex	
Male	225 (48.8%)
Female	236 (51.2%)
Site	
Distal Radius	54 (11.7%)
Proximal Femur	25 (5.4%)
Distal Femur	153 (33.2%)
Proximal Tibia	120 (26.0%)
Distal Tibia	17 (3.7%)
Proximal Humerus	23 (5.0%)
Others	69 (15.0%)
Campanacci Classification	
Stage I	9 (2.0%)
Stage II	269 (58.4%)
Stage III	183 (39.7%)
Previous Surgery	
None	383 (83.1%)
1	78 (16.9%)
Surgery	
Curettage	270 (58.6%)
Resection or Amputation	191 (41.4%)
Local Recurrence	
None	378 (82.0%)
1	63 (13.7%)
≥2	20 (4.3%)
Lung Metastasis	
None	431 (93.5%)
Synchronous	9 (2.0%)
Metachronous	21 (4.6%)
Malignant Transformation	
None	446 (96.7%)
1	15 (3.3%)
Follow-Up (Months)	
Median	89.4
Interquartile Range	54.8–134.0

**Table 2:** Details of 15 patients who experienced malignant transformation from benign GCTB.

Case	Sex	Age	Site	Campanacci Stage	Lung Metastasis at Presentation	Previous Surgery	Surgery	Total Number of Local Recurrence
1	M	63	Proximal femur	Stage 3	No	No	Resection	1
2	F	21	Distal femur	Stage 2	Yes	No	Resection	0
3	F	27	Proximal tibia	Stage 2	No	No	Curettage	1
4	M	31	Proximal tibia	Stage 2	No	No	Curettage	0
5	M	34	Proximal tibia	Stage 2	No	No	Curettage	6
6	F	36	Distal ulna	Stage 2	No	No	Curettage	1
7	M	31	Distal femur	Stage 2	Yes	No	Curettage	0
8	M	24	Proximal tibia	Stage 2	No	No	Curettage	1
9	M	26	Proximal tibia	Stage 2	No	No	Curettage	1
10	M	37	Distal femur	Stage 2	No	No	Curettage	2
11	M	77	Distal femur	Stage 2	No	No	Curettage	1
12	M	42	Proximal tibia	Stage 2	No	No	Curettage	1
13	M	47	Proximal femur	Stage 2	No	No	Curettage	1
14	M	72	Proximal tibia	Stage 3	No	No	Curettage	1
15	F	62	Distal radius	Stage 3	No	No	Curettage	1

GCTB, giant cell tumor of bone.

**Table 3:** Details of 15 patients who experienced malignant transformation from benign GCTB.

Case	Histology of Malignant GCTB	H3F3A G34W Mutation on Sarcomatous Component	Interval between Initial Surgery of Benign GCTB and Malignant Transformation (Years)	Interval between Last Surgery for Benign GCTB and Local Recurrence with Malignant Transformation (Years)	Distant Metastases at Diagnosis of Malignant GCTB	Treatment for Malignant GCTB	Status	Follow-Up Period from Diagnosis of Malignant GCTB (Months)
1	UPS	Negative	6.5	6.5	No	External hemipelvectomy	DOD	19
2	Osteosarcoma	Negative	11.4	NA	Yes	Palliative CHT	AWD	9
3	Osteosarcoma	Negative	22.8	22.8	No	Neo- and adjuvant CHT, resection	NED	46
4	UPS	Negative	13.7	NA	No	Neo- and adjuvant CHT, amputation	NED	33
5	Osteosarcoma	Negative	32	26.3	No	Neo- and adjuvant CHT, resection	NED	97
6	Osteosarcoma	Negative	1.7	1.7	No	Neo- and adjuvant CHT, resection	NED	86
7	Osteosarcoma	Negative	26.3	NA	Yes	Palliative CHT	AWD	9
8	UPS	Positive	4.8	4.8	No	Amputation and adjuvant CHT	NED	10
9	UPS	Positive	4.1	4.1	No	Resection	NED	66
10	UPS	Negative	16	14	No	Disarticulation, adjuvant CHT	NED	123
11	Osteosarcoma	Negative	27	27	Yes	Palliative RT	DOD	3
12	Osteosarcoma	Negative	16.3	16.3	No	RT, amputation	DOOD	122
13	Osteosarcoma	Negative	22	22	Yes	Palliative RT, CHT	DOD	2
14	Osteosarcoma	Negative	27.7	27.7	No	Amputation	NED	113
15	Osteosarcoma	Negative	7.3	7.3	No	Resection, adjuvant CHT	NED	32

GCTB, giant cell tumor of bone; UPS, undifferentiated pleomorphic sarcoma; NED, no evidence of disease; AWD, alive with disease; DOD, dead of disease; DOOD, dead of other disease; CHT, chemotherapy; RT, radiotherapy; NA, not applicable.

**Table 4:** Univariate analysis for malignant transformation-free survival in the patients with GCTB.

Variable	No. of Patients (*n* = 461)	10-Year Malignant Transformation-Free Survival (95% CI) (%)	*p*-Value
Age (Years)			0.806
<30	225	98.9 (95.5–99.7)	
30≤	236	97.9 (93.6–99.4)	
Sex			0.398
Male	225	98.0 (94.0–99.4)	
Female	236	98.7 (94.8–99.7)	
Site			0.967
Distal Radius/Proximal Femur	79	96.1 (85.8–99.0)	
The Others	382	99.0 (97.0–99.7)	
Campanacci Classification			0.575
Stage I, II	278	98.6 (95.8–99.6)	
Stage III	183	98.0 (92.2–99.5)	
Previous Surgery			0.117
None	383	98.1 (95.3–99.2)	
1	78	100.0	
Surgery			0.042 *
Curettage	270	97.8 (94.2–99.2)	
Resection or Amputation	191	99.2 (94.3–99.9)	
Local Recurrence			0.002 *
None	378	100.0	
1	63	92.4 (81.2–97.1)	
≥2	20	92.3 (60.9–98.9)	
Lung Metastasis			0.751
None	431	98.3 (95.8–99.3)	
Synchronous or Metachronous	30	100.0	

* Statistically significant (*p* < 0.05).

**Table 5:** Multivariable cox regression analysis of malignant transformation-free survival in the patients with GCTB.

Variable	Hazard Ratio (95% CI)	*p*-Value
Local Recurrence		
1 Versus None	11.33 (2.33–55.13)	0.003 *
≥2 Versus None	11.24 (1.76–71.96)	0.011 *

* Statistically significant (*p* < 0.05).

## Data Availability

The datasets generated, analyzed, or both during the present study are not publicly available because of privacy issues but are available from the corresponding author upon reasonable request.

## Supplementary Materials

The following are available online at https://www.mdpi.com/article/10.3390/cancers13143644/s1, Table S1: Characteristics of patients excluded from this study because they received denosumab. Table S2: Details of two patients who received surgery combined with denosumab treatment and experienced malignant transformation, Table S3: Details of two patients who received surgery combined with denosumab treatment and experienced malignant transformation. In the both patients, the H3F3A mutation was negative.
