# Supplementary material for: Late Local Recurrence of Bone Giant Cell Tumors Associated with an Increased Risk for Malignant Transformation
Source: Cancers (Basel). 2021 Jul 20;13(14):3644. doi: 10.3390/cancers13143644 (PMC8304778; doi:10.3390/cancers13143644)
Supplement: Supplementary file 1 [file cancers-13-03644-s001.zip › cancers-1287549-supplementary.pdf]

Supplementary Materials

Table S1. Details of the 26 patients who experienced lung metastases without malignant transformation.

Case	Age	Sex	Location of tumor	Campanacci classification	Previous surgery	Lung metastasis at presentation	Surgery for primary tumor	Pre- and postoperative denosumab treatment	Local recurrence	Interval between surgery of primary tumor and discovery of lung metastasis (months)	Treatment for lung lesions	Follow-up period from surgery for primary tumor (months)	Status
1	32	F	Distal femur	Stage 3	No	Yes	Resection	No	No	0	Observation	34	AWD
2	26	F	Distal radius	Stage 3	No	Yes	Resection	No	Yes	0	Metastasectomy	42	AWD
3	35	M	Proximal tibia	Stage 2	Yes	No	Resection	No	No	124	Observation	400	AWD
4	47	M	Distal femur	Stage 3	No	No	Resection	No	No	43	Observation	173	AWD
5	20	F	Distal radius	Stage 3	No	No	Resection	No	No	35	Observation	89	AWD
6	24	F	Distal radius	Stage 3	No	No	Resection	No	Yes	11	Metastasectomy	139	NED
7	54	F	Phalanx	Stage 2	No	No	Resection	No	Yes	22	Observation	42	AWD
8	51	F	Distal radius	Stage 3	Yes	Yes	Resection	No	Yes	0	Observation	134	AWD
9	63	M	Proximal femur	Stage 2	No	No	Resection	No	Yes	35	Metastasectomy	97	AWD
10	27	F	Proximal tibia	Stage 3	No	No	Resection	No	No	24	Metastasectomy	181	NED
11	27	M	Proximal humerus	Stage 3	No	No	Resection	No	No	11	Metastasectomy	136	AWD

12	39	M	Proximal fibula	Stage 3	Yes	Yes	Amputation	No	No	0	Metastasectomy	66	DOOD
13	17	F	Proximal tibia	Stage 3	No	Yes	Resection	No	No	0	Chemotherapy and denosumab therapy	118	AWD
14	33	M	Proximal tibia	Stage 3	Yes	No	Resection	No	No	64	Observation	114	AWD
15	23	F	Distal femur	Stage 2	No	No	Curettage	No	No	34	Metastasectomy	81	NED
16	38	F	Distal femur	Stage 2	Yes	No	Resection	No	No	11	Metastasectomy	141	NED
17	28	F	Metacarpal	Stage 3	No	No	Resection	No	Yes	30	Metastasectomy	118	NED
18	22	M	Distal radius	Stage 3	No	No	Resection	No	Yes	114	Metastasectomy	223	NED
19	31	F	Metatarsal	Stage 3	Yes	Yes	Resection	No	No	0	Observation	71	AWD
20	32	M	Metacarpal	Stage 3	No	No	Resection	No	No	23	Observation	76	AWD
21	37	M	Proximal tibia	Stage 3	No	No	Curettage	No	No	46	Observation	120	AWD
22	24	F	Proximal fibula	Stage 3	No	No	Resection	No	Yes	44	Observation	73	AWD
23	25	M	Proximal tibia	Stage 2	No	No	Curettage	No	Yes	10	Observation	150	AWD
24	28	F	Distal humerus	Stage 3	Yes	Yes	Curettage	No	No	0	Metastasectomy	183	NED
25	27	M	Distal radius	Stage 3	Yes	No	Resection	No	Yes	292	Metastasectomy	312	NED
26	18	M	Distal radius	Stage 3	No	No	Resection	No	No	17	Observation	63	DOD

NED, no evidence of disease; AWD, alive with disease; DOD, death of disease; DOOD, death of other disease

Table S2. Characteristics of patients excluded from this study because they received denosumab.

**Variable (*n* = 36)**	**No. of patients**
Age (years)	
Median	36.8
Interquartile range	26.0-45.2
Sex	
Male	16 (44.4%)
Female	20 (55.6%)
Site	
Distal radius	13 (36.1%)
Proximal femur	1 (2.8%)
Distal femur	5 (13.9%)
Proximal tibia	7 (19.4%)
Distal tibia	2 (5.6%)
Proximal humerus	5 (13.9%)
Others	3 (8.3%)
Campanacci classification	
Stage II	16 (44.4%)
Stage III	20 (55.6%)
Previous surgery	
None	30 (83.3%)
1	6 (16.7%)
Surgery	
No surgery	1 (2.8%)
Curettage	30 (83.3%)
Resection	5 (13.9%)
Local recurrence	
None	15 (41.7%)
1	14 (38.9%)
≥2	7 (19.4%)
Lung metastasis	
None	31 (86.1%)
Synchronous	1 (2.8%)
Metachronous	4 (11.1%)
Malignant transformation	

Table S3. Details of two patients who received surgery combined with denosumab treatment and experienced malignant transformation.

Case	Sex	Age	Site	Campanacci stage	Lung metastasis at presentation	Previous surgery	Denosumab treatment	Surgery
16	M	64	Distal radius	Stage 3	No	No	Yes*	None
17	F	71	Proximal humerus	Stage 3	No	No	Yes	Resection

Case	Total number of local recurrences	Histology of malignant GCTB (H3F3A G34W mutation)	Interval between initial surgery of benign GCTB and malignant transformation (years)	Interval between last surgery of benign GCTB and local recurrence with malignant transformation (years)	Distant metastases at diagnosis of malignant GCTB	Treatment for malignant GCTB	Status	Follow-up period from diagnosis of malignant GCTB (months)
16	1	UPS (negative)	8.3	8.3	Yes	Amputation	AWD	2
17	2	Osteosarcoma (negative)	4	4	Yes	Palliative CHT, amputation	DOD	16

GCTB, giant cell tumor of bone; UPS, undifferentiated pleomorphic sarcoma; AWD, alive with disease; DOD, dead of disease; CHT, chemotherapy*Denosumab treatment alone.
